# Planet Microbe: a platform for marine microbiology to discover and analyze interconnected ‘omics and environmental data

**DOI:** 10.1093/nar/gkaa637

**Published:** 2020-07-31

**Authors:** Alise J Ponsero, Matthew Bomhoff, Kai Blumberg, Ken Youens-Clark, Nina M Herz, Elisha M Wood-Charlson, Edward F Delong, Bonnie L Hurwitz

**Affiliations:** Department of Biosystems Engineering, University of Arizona, Tucson, AZ, USA; BIO5 Institute, University of Arizona, Tucson, AZ, USA; Department of Biosystems Engineering, University of Arizona, Tucson, AZ, USA; BIO5 Institute, University of Arizona, Tucson, AZ, USA; Department of Biosystems Engineering, University of Arizona, Tucson, AZ, USA; BIO5 Institute, University of Arizona, Tucson, AZ, USA; Department of Biosystems Engineering, University of Arizona, Tucson, AZ, USA; BIO5 Institute, University of Arizona, Tucson, AZ, USA; Department of Biosystems Engineering, University of Arizona, Tucson, AZ, USA; Environmental Genomics and Systems Biology Division, E.O. Lawrence Berkeley National Laboratory, Berkeley, CA, USA; Daniel K. Inouye Center for Microbial Oceanography: Research and Education, University of Hawaii, Manoa, Honolulu, HI 96822, USA; Department of Biosystems Engineering, University of Arizona, Tucson, AZ, USA; BIO5 Institute, University of Arizona, Tucson, AZ, USA

## Abstract

In recent years, large-scale oceanic sequencing efforts have provided a deeper understanding of marine microbial communities and their dynamics. These research endeavors require the acquisition of complex and varied datasets through large, interdisciplinary and collaborative efforts. However, no unifying framework currently exists for the marine science community to integrate sequencing data with physical, geological, and geochemical datasets. Planet Microbe is a web-based platform that enables data discovery from curated historical and on-going oceanographic sequencing efforts. In Planet Microbe, each ‘omics sample is linked with other biological and physiochemical measurements collected for the same water samples or during the same sample collection event, to provide a broader environmental context. This work highlights the need for curated aggregation efforts that can enable new insights into high-quality metagenomic datasets. Planet Microbe is freely accessible from https://www.planetmicrobe.org/.

## INTRODUCTION

Oceanographic research cruises produce large quantities of data using a wide range of methods and equipment that require large collaborative efforts. These research endeavors span a broad range of disciplines and are critical to investigating the spatiotemporal interplay between biological, geological and chemical processes in marine systems. Importantly, the advent of genomic sequencing technologies has allowed for greater insight into the distribution and dynamics of microbial populations in marine ecosystems. In 2004, the Global Ocean Survey (GOS) launched the first large-scale oceanic sequencing expedition that led to the identification of a large number of novel genes in the ocean (1). More recently, the TARA ocean expedition (2), an international sequencing effort, revealed surprisingly high biodiversity in the oceans and identified novel interactions between oceanic microorganisms.

Despite scientists’ best efforts to carefully curate and share their data with collaborators to advance individual studies and publications, no systematic, unifying framework currently exists to integrate ‘omics data with physical, geochemical and biological datasets commonly used by the broader geoscience community. As a result, the moment each sample leaves the ship is often the last time each data component appears together in a unified collection. Typically, ‘omics datasets are submitted to nucleotide sequence repositories like the Sequence Read Archive (SRA) (3), whereas contextual environmental data are submitted and stored in specialized data-repositories (such as the Biological and Chemical Oceanography Data Management Office (BCO-DMO) (4) or PANGEA), or only made available within published papers. This makes it difficult to fully reconnect in-situ data from the same sampling event. The development of resources to facilitate the aggregation and publication of biological datasets along with their physicochemical information is critical for studying marine microbes and the biogeochemical processes in the ocean that they drive.

In 2011, the SRA began integrating the BioProject and BioSample databases from NCBI (5) to ensure that sequence data are accompanied by a minimum set of information. This Minimum Information about any (x) nucleotide Sequence (MIxS) is a unified standard developed by the Genomic Standards Consortium (6). Although the majority of the MIxS descriptors are optional, the standard mandates the annotation of sequence data with information about the collection date and localization, as well as the description of the biome, environmental feature and material from which the sample was collected (6). Despite these efforts, the quantity and quality of the contextual data provided within genomic submissions are highly variable. In 2018, the MGnify web-portal (formerly known as EBI-Metagenomics) was released, allowing users to search for metagenomes using several contextual data attributes, such as collection depth, biome or temperature (7). However, searching these ‘omics datasets by their accompanying information is impaired by a lack of consistent terminology as well as mislabeled or missing contextual data accompanying metagenomes, limiting their reuse in meta-analyses. Recently, domain scientists have rallied to improve and curate standards within their fields. For example, Bernstein *et al.* curated and standardized data for human-associated samples and metagenomes deposited in SRA (8). Similarly, the TerrestrialMetagenomeDB collected and curated terrestrial metagenomic datasets (9). However, these projects only include data from sequencing repositories (e.g. SRA (3), MG-RAST (10) and EBI (11)) without integrating contextual data stored in published papers or other environmental data repositories. Similar efforts exist for genomic data, such as the MAR databases that aims to collect reference genomes (MarRef), including both complete (MarRef) and incomplete marine prokaryotic genomes (MarDB) or marine fungi (MarFun) (12). The MAR database project integrates data from several sources of information including sequence, taxonomy and literature databases to describe their genomic datasets. Similarly, Pasolli *et al.* developed a pipeline to integrate different sources of contextual and taxonomic data for human-associated samples deposited in SRA (13). These datasets were made accessible to users through the R package ExperimentHub. For polar ecosystems, the Microbial Antarctic Resource system (mARS) aims to facilitate the discovery, access, and analysis of molecular microbial diversity data generated by Antarctic researchers (14). Importantly, these resources allow access to ‘omics resources using curated contextual data but do not provide computing resources to users to further analyze these datasets. On the other hand, large oceanic gene catalogs constructed from metagenomes, such as the Ocean Gene Atlas, allow users to identify gene abundance co-variation with marine environmental variables (15). However, these gene catalogs are typically constructed from a single sequencing expedition. A notable exception is the MAR databases that provide a gene catalog constructed on both the Tara Oceans and Ocean Sampling Day (OSD) datasets. Additionally, the MAR database provides a BLAST service to query across their different collections (16).

Here, we present Planet Microbe, a web-based portal for the open sharing and discovery of historical and ongoing oceanographic sequencing efforts. Planet Microbe integrates historical oceanographic ‘omics datasets (Hawaii Ocean Time-series (HOT) (17–21), Bermuda Atlantic Time-series (BATS) (22), Global Ocean Sampling Expedition (GOS) (23), Amazon continuum dataset (ANACONDAS) (24,25) and Center for Dark Energy Biosphere Investigations (C-DEBI) (26)) along with datasets from large-scale ocean expeditions such as the TARA Oceans (27) and Arctic Expeditions (28) and Ocean Sampling Day (OSD) (29). In Planet Microbe, these ‘omics data have been reintegrated with their in-situ environmental contextual data, including biological and physicochemical measurements, and information about sampling events, and sampling stations. Finally, cruise tracks, protocols, and instrumentation are also linked to these datasets to provide users with a comprehensive view of the metadata. Additionally, Planet Microbe integrates computational tools using National Science Foundation (NSF) funded Cyberinfrastructure (CyVerse) and provides users with free access to large-scale computing power to analyze and explore these datasets (30,31).

## RESULTS

### Database content and platform functionality

#### Database content overview

**Figure 1.**
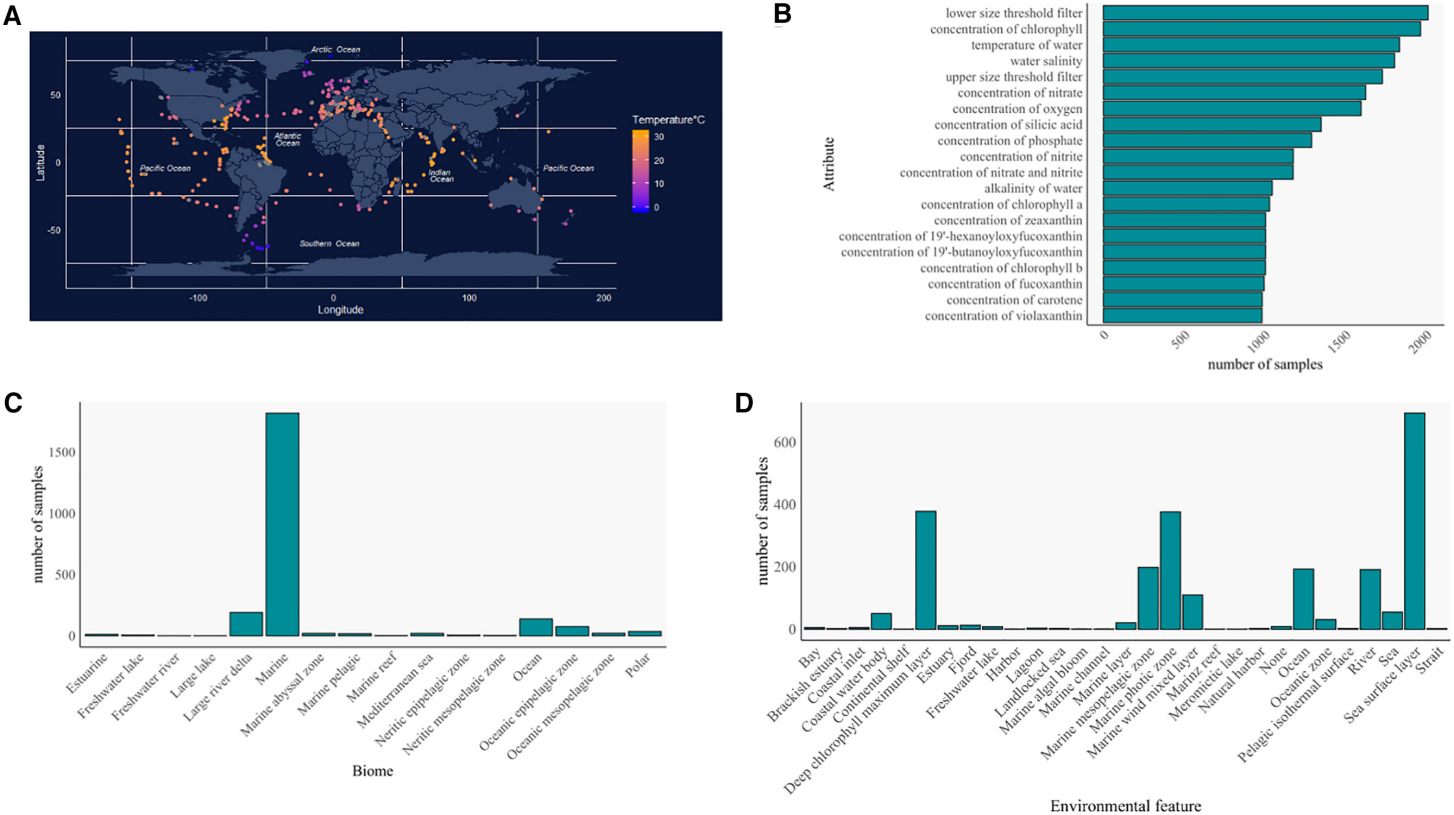
Overview of the Planet Microbe database content. (**A**) Map of the samples in the database. Samples are color-coded by temperature with samples missing temperature values shown in gray. (**B**) Distribution of the 20 most abundant sample attributes in Planet Microbe datasets, excluding the mandatory attributes of latitude, longitude, depth and time. (**C**) Distribution of ENVO biome classes associated with samples. (**D**) Distribution of the ENVO environmental feature classes associated with samples in Planet Microbe.

This first release of Planet Microbe includes 2371 aquatic samples collected from multiple projects, encompassing >10 years of experiments, the oldest of which was taken as part of the HOT project in 2007. The majority of samples in this database release are from the Tara Ocean or Tara Polar expedition (54%), HOT time-series (24%) and OSD (7%) given the large extent of sampling for each of these projects. Of these samples, 57% have metagenome(s), 27% have amplicon sequencing dataset(s) and 16% have metatranscriptome(s) (random or PolyA amplified). Most of the samples in Planet Microbe are from surface water, with 45% of the samples collected between 0 and 10 m depth. Planet Microbe contains samples collected from all five oceans and most seas (Figure 1A). While the majority of the samples in Planet Microbe were collected in a marine biome, samples are available for 16 aquatic biome types in total (Figure 1C and D).

In total, the terminology for 108 contextual sample attributes were standardized, with each investigator's original naming for these attributes mapped to terms from the Planet Microbe application ontology. Of those, 87 are searchable through the Planet Microbe search interface. To be integrated into Planet Microbe, samples must have a minimum of seven attributes (collection date, latitude, longitude, and depth, as well as biome, environmental feature and material). Aside from these required attributes, most samples also include water temperature, salinity, oxygen concentration and chlorophyll concentration (Figure 1B).

#### Search and download datasets of interest

**Figure 2.**
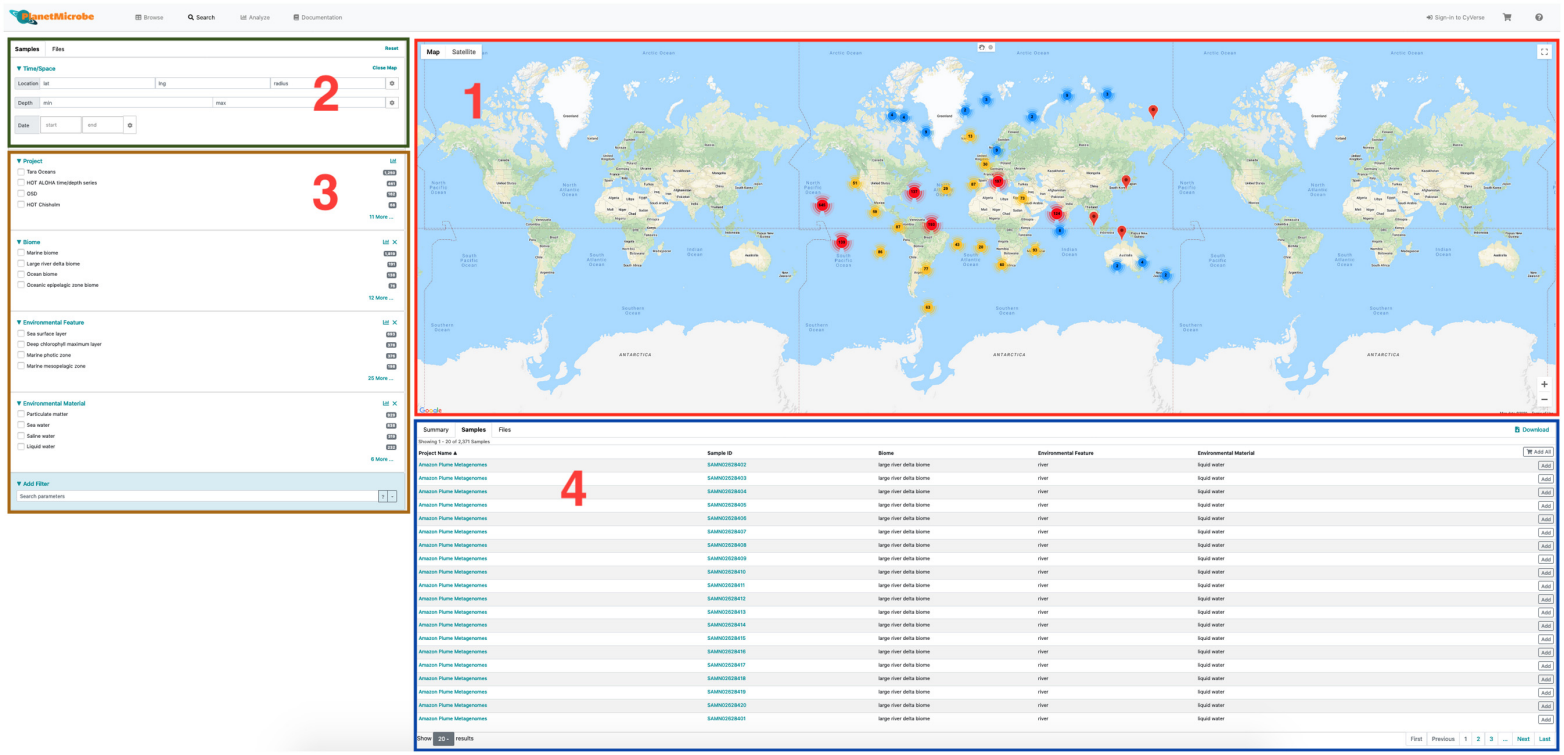
Planet Microbe search interface. The Search interface is divided in four areas: (1) The map search allows users to navigate the map for geographical regions of interest using the hand tool or to use the circular selection tool to designate a region in which to select samples. (2) The top left panel of the search interface allows users to select samples based on their mandatory four dimensions (latitude, longitude, depth and collection time). (3) The sample attribute search allows users to further refine their search using a set of curated attribute terms. (4) The result of the search is displayed in the result area, and the samples and attributes used for the search are listed in a table in the ‘samples’ panel.

In Planet Microbe, the ‘Search’ tab constitutes the main search interface for samples present in the database (Figure 2). This interface allows users to search for and select samples based on the sample's contextual data or the ‘omic experiment description.

Through the sample search interface, a map provides an intuitive way of selecting samples directly from their geographical localization. Moreover, the top left of the search interface allows users to select samples across four dimensions (latitude, longitude, depth and collection time). Finally, the sample attribute search allows users to further refine their search, selecting samples based on their Biome, environmental feature and material, as well as a set of 87 curated terms. Search results are displayed as a table and summary bar charts. Search results are available for download as a tab-delimited file and can be selected and saved in a personal cart for further analysis.

The search can be further refined using the ‘File’ search tab, allowing users to filter the ‘omics data on the SRA run attributes. These attributes describe the library construction method and the sequencing strategy.

### Analytic capabilities

Planet Microbe offers capabilities to run computational tools (called ‘Apps’) on samples that users add to their cart. Additionally, users can run these Apps on private datasets stored in the CyVerse Data Store. A free CyVerse account is required to run Apps. The first release of Planet Microbe has four Apps integrated: Centrifuge, a read-based taxonomic annotation tool (32); two *de-novo* comparative metagenomic tools, Libra (33) and MASH (34); and an App that runs BLAST (16) against the ALOHA gene catalog, a metagenomic survey of microbes collected by the HOT program (17,19,21).

### Reintegrating samples in their broader context

The dynamics and composition of microbial populations in ‘omics datasets are best understood in context with environmental factors associated with the original water sample, including temperature, depth, and other physicochemical and biological properties. Yet, interlinking ‘omics data with environmental data derived from water samples can be difficult given variations in the collection processes employed by oceanographic expeditions. For example, sampling events associated with Niskin bottles mounted to a frame can collect samples from multiple depths in the water column, whereas net tows collect biological material from the same depth (or across depth intervals), across horizontal transects of varying distances. Similarly, metagenomic samples from time-sequencing sediment traps collect sinking particulate material at a fixed geospatial point across varying time intervals (35), whereas free-drifting sediment traps sample across variable spatiotemporal intervals (36).

**Figure 3.**
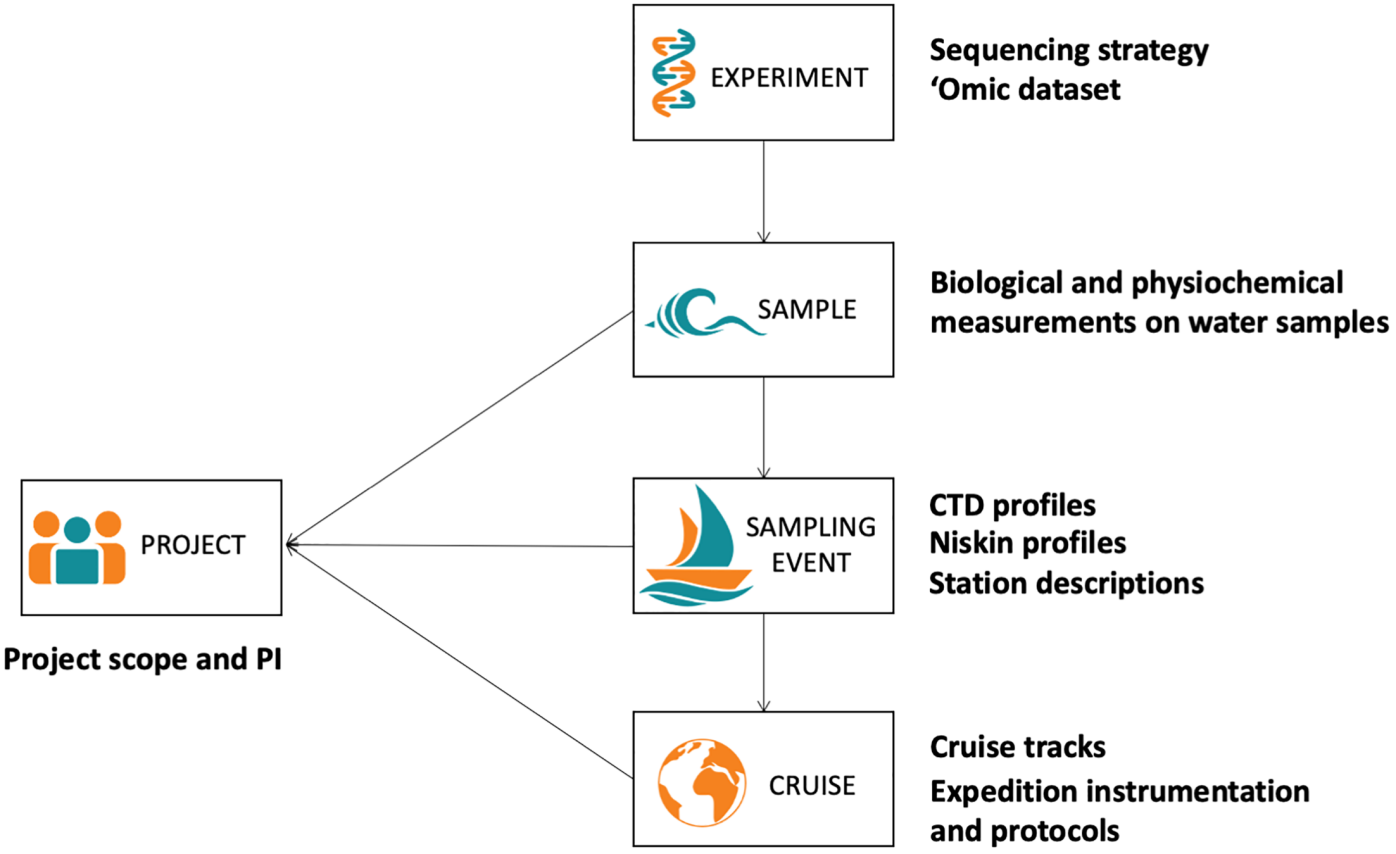
Planet Microbe data model. In Planet Microbe, the data model allows users to link ‘omics data to the environmental context from the sample, sampling event and sampling expedition. Users can also search for all samples that are associated with a single project, even if they were collected by different project leaders.

To account for variations in sampling protocols, Planet Microbe uses a data model (Figure 3) that links ‘omics experiments, with samples, sampling events, and collection campaigns. At a finer scale, the ‘omics experiment description encompasses information about the number of runs, the sequencing technology and methods used for the ‘omics library construction. In Planet Microbe, all ‘omic experiments are linked to one or more samples. The sample page aggregates all biological and physio-chemical information collected from these samples (e.g. https://www.planetmicrobe.org/#/samples/33). Sample and contextual data taken during the same sampling event can be found by exploring the sampling event page (e.g. https://www.planetmicrobe.org/#/sampling_events/19). Finally, the cruise page links all sampling events as well as any additional cruise information (e.g. https://www.planetmicrobe.org/#/campaigns/2). Projects in Planet Microbe allow users to explore samples, sampling events, and cruises that are part of the same project and produced by a unique investigator or a team of investigators (e.g. https://www.planetmicrobe.org/#/projects/1).

### Exploring multi-projects sampling efforts: the HOT dataset

Since October 1988, the HOT program, based out of the University of Hawai‘i at Mānoa, has conducted roughly monthly monitoring and sampling at Station ALOHA, ∼100 km north of O‘ahu, Hawai‘i. This program aims to provide a comprehensive biological, physicochemical, and hydrological description of the North Pacific subtropical gyre across time (19).

In NCBI, the different HOT ‘omics datasets are separated into three distinct BioProjects, according to their lead principal investigator, collection date and sequencing technology used when the data were generated. This separation prevents simple connections between the different sampling efforts led during HOT sampling campaigns. In Planet Microbe, the data model allows users to reconnect these projects and retrieve all samples taken during a sampling cruise or specific sampling event, even if they were produced by different teams. For example, cruise HOT214 contains three ‘omics samples taken in two distinct projects (HOT-Delong and HOT-Chisolm) that can easily be reconnected and retrieved through this data model (https://www.planetmicrobe.org/#/campaigns/57).

Another benefit of the Planet Microbe data model is the ability to connect and store additional contextual data at the sampling event or sampling campaign level. For example, during cast no. 1 of the cruise HOT232, (https://www.planetmicrobe.org/#/sampling_events/136) two samples were taken (at depth 25 and 75 m). Importantly, a conductivity–temperature–density (CTD) profile was measured during collection, and several additional measurements were performed on water collected at different depths during this sampling event. Although these measurements were not taken from the exact same water samples as the metagenomic samples, measurements within close proximity can be used to describe the broader context in which the microbial population was observed. Consequently, in Planet Microbe, the complete CTD profile and all measurements taken during the cast are made available in the sampling event page.

### Integration of complementary data sources

#### Various information sources augment sample descriptions

Planet Microbe brings together information from various data sources (NCBI, BCO-DMO, PANGEA etc.) to ensure the description of the sample's contextual environment is as comprehensive as possible. For example, the BATS sample SAMN07137101 is described by 10 attributes in NCBI, but using information from BCO-DMO, Planet Microbe contains a total of 68 attributes for this sample, 41 of which are searchable fields through the Planet Microbe search interface. Although this strategy leads to some data redundancy by integrating different sources of information in Planet Microbe, it also allows for greater resilience against mistakes and forgotten objects. For example, the HOT sample SAMN05991668 contains 19 terms in NCBI, however, 10 of them were submitted without units. Because this contextual information was also published as supplemental material in a published paper, the sample is available in Planet Microbe without information loss. In order to allow users to refer back to the original data source, the source URL or Digital Object Identifier (DOI) is displayed for each datum in Planet Microbe.

#### Search examples across different data sources

Planet Microbe leverages an application ontology to harmonize the terminology describing attributes that come from various data sources. This terminological harmonization allows users to search for samples using standardized sample descriptors which are mapped to the original attributes from different data sources. Importantly, the search in Planet Microbe can be performed on an array of data from different sources. For example, if a sample contains three temperature measurements, including some variations (temperature reported using different methods or at different precision), all these measurements will be discovered by the search.

## MATERIALS AND METHODS

### Database construction

#### Overview

**Figure 4.**
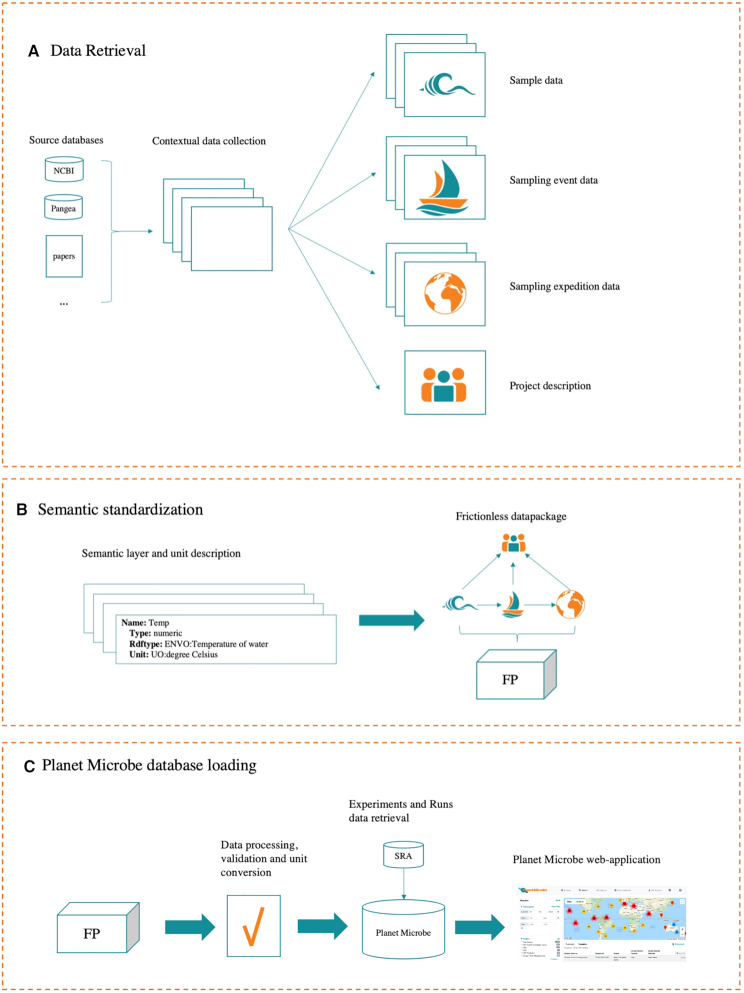
Overview of the Database construction. (**A**) Contextual data is retrieved from various databases and published papers. The data is divided according to the Planet Microbe data model into various elements (sample, sampling event, sampling expedition and project) (**B**) Terminological standardization of attributes. Sample attributes are mapped with type and unit descriptions allowing their standardization between projects. All contextual data describing samples, sampling events and sampling expeditions were then assembled into a Frictionless Data package (FP). (**C**) Finally, datasets were uploaded into Planet Microbe. The type and format of the contextual data is reviewed and units are converted to standard recommended units. SRA Experiment and Run information are retrieved and made accessible through the Planet Microbe web interface.

Planet Microbe datasets were constructed as follows and summarized in Figure 4. First, contextual data about the samples and projects were collected from the NCBI BioSample and BioProject databases and then parsed and curated. Additional contextual data about samples were then collected from other repositories and published papers. In Planet Microbe, we defined the sampling event object as the broader context in which samples were collected. In most genomic repositories, the contextual data describing samples (other in-situ measurements that were taken from the water sample used to generate ‘omics data) is mixed with data concerning sampling events (a description of the water column and oceanographic station in which a sample was taken). In Planet Microbe, these two distinct types of contextual information were parsed and separated. Finally, additional information about sampling expeditions was collected from cruise repositories (e.g. the Rolling Deck Repository). All contextual data describing samples, sampling events, and sampling expeditions were then assembled into a Frictionless Data package (http://frictionlessdata.io/).

Frictionless Data is a technical standard for the containerization, publication, and mobilization of data. Frictionless Data provides specifications and software libraries for the construction and use of Frictionless Data Packages. Frictionless Data Packages are Javascript Object Notation Format (JSON) files in which metadata about multiple data resources such as comma-separated value files (CSV) can be encapsulated. Frictionless Data Packages have previously been used to encapsulate and annotate plant metabolomics data (37). In Planet Microbe, we make use of Frictionless Data Packages to bring together multiple CSV resource files and annotate their individual data attributes with additional metadata within a master Data Package JSON file. The Frictionless Data packages produced for Planet Microbe are freely accessible in GitHub (https://github.com/hurwitzlab/planet-microbe-datapackages).

Finished data packages were loaded into a Postgres database and the information about the Experiments and Runs associated with the samples were taken from the SRA database.

#### Public data resources utilized for the construction of Planet Microbe

In order to construct the Planet Microbe database, information from a number of resources were used and made accessible through Planet Microbe. The list of resources used for the project is listed in Table 1 and the list of projects included in the first release are listed in Table 2.

**Table 1.** Public data resources utilized for the construction of Planet Microbe

Resource Type	Name	URL
Sequence databases	NCBI, National Center for Biotechnology Information	ncbi.nlm.nih.gov
	ENA, European Nucleotide Archive	ebi.ac.uk/ena
	SRA, Sequence Read Archive	trace.ncbi.nlm.nih.gov/Traces/sra
Contextual databases	BCO-DMO, Biological, and Chemical Oceanography Database Management Office	bco-dmo.org
	PANGEA, Data Publisher for Earth and Environmental sciences	pangaea.de
	R2R, Rolling Deck repository	rvdata.us
	CCHDO	cchdo.ucsd.edu
	SAMOS, Shipboard Automated Meteorological, and Oceanographic System	samos.coaps.fsu.edu
	WHOI, Data Library and Archive	dla.whoi.edu
	IEDA, Marine Data Science Geosystem	marine-geo.org
Literature databases	PubMed	ncbi.nlm.nih.gov/pubmed
	Doi, Digital Object Identifier System	doi.org

**Table 2.** Datasets included in the database

Dataset name	Dataset description	Expedition	NCBI ID	# Samples	Resources used
Chisholm HOT	Temporal sampling of marine metagenomes from Station ALOHA	HOT	385855	68	NCBI, BCO-DMO, R2D, CCHDO
DeLong HOT metagenomes	Temporal sampling of marine metagenomes from Station ALOHA	HOT	16339	42	NCBI, BCO-DMO, R2D, CCHDO, published papers
OSD2014	Amplicon and metagenome samples from the 2014 Ocean Sampling Day event around the world	OSD	276999	162	NCBI, Pangea
HOT Delong metatranscriptome	Temporal sampling of marine metatranscriptomes from Station ALOHA	HOT	16339	8	NCBI, BCO-DMO, R2D, CCHDO, published paper
HOT ALOHA time/depth series	Temporal sampling of marine metagenomes from Station ALOHA collected from 2010 to 2016	HOT	352737	461	NCBI, BCO-DMO, R2D, CCHDO, SAMOS, published paper
GOS expedition 2009–2011	Samples from the Global Ocean Survey expedition (2009-2011). Include coastal sites in the Pacific and Atlantic Ocean, Mediterranean, Baltic and Black Sea as well as some large lakes.	GOS	293636	68	NCBI
AHX	Shotgun Sequencing of Tara Oceans DNA samples corresponding to size fractions for protist	Tara Oceans	213098	651	NCBI, Pangea
ANB	Shotgun Sequencing of Tara Oceans DNA samples corresponding to size fractions for large DNA viruses	Tara Oceans	196958	75	NCBI, Pangea
APX	Shotgun Sequencing of Tara Oceans DNA samples corresponding to size fractions for small DNA viruses	Tara Oceans	214077	92	NCBI, Pangea
APY	Shotgun Sequencing of Tara Oceans DNA samples corresponding to size fractions for prokaryotes	Tara Oceans	196960	136	NCBI, Pangea
ARC	Metatranscriptome sequencing from samples corresponding to size fractions for protists	Tara Oceans	253559	23	NCBI, Pangea
ARD_2	Metatranscriptome sequencing from samples corresponding to size fractions for protists	Tara Oceans	253564	450	NCBI, Pangea
ARG	Amplicon sequencing of Tara Oceans DNA samples corresponding to size fractions for protists	Tara Oceans	253565	883	NCBI, Pangea
ARH	Amplicon sequencing of Tara Oceans RNA samples corresponding to size fractions for protists	Tara Oceans	261507	14	NCBI, Pangea
ARI	Amplicon sequencing of Tara Oceans DNA samples corresponding to size fractions for prokaryotes or protist	Tara Oceans	213100	7	NCBI, Pangea
AUZ	Amplicon sequencing of Tara Oceans DNA samples corresponding to size fractions for large DNA viruses	Tara Oceans	253560	19	NCBI, Pangea
BEZ	Metatranscriptome sequencing of Tara Oceans DNA samples corresponding to size fractions for prokaryotes	Tara Oceans	253563	6	NCBI, Pangea
BNA	Shotgun Sequencing of Tara Oceans Polar Circle DNA samples corresponding to size fractions for small DNA viruses	Tara Polar	288560	41	NCBI, Pangea
BATS_Chisholm	Temporal sampling of marine metagenomes from the Bermuda Atlantic Time series	BATS	385855	62	NCBI, BCO-DMO, R2R
C-DEBI mid-ocean ridge flank	Metagenomic samples from deep seawater microbial communities	C-DEBI	268250 and 266365	20	NCBI, BCO-DMO, published paper, R2R, SAMOS, WHOI, IEDA
Amazon Plume Metagenomes	Metagenomic samples from the Amazon river-sea continuum sampling effort. Samples from the Amazon Plume.	ANACONDAS	237344	48	NCBI, BCO-DMO, R2R, SAMOS, WHOI
Amazon River Metagenomes	Metagenomic samples from the Amazon river-sea continuum sampling effort. Samples from the Amazon river.	ANACONDAS	237344	48	NCBI
Amazon Plume Metatranscriptomes	Metatranscriptomic samples from the Amazon river-sea continuum sampling effort. Samples from the Amazon plume.	ANACONDAS	237345	34	NCBI, BCO-DMO, R2R, SAMOS, WHOI
Amazon River Metatranscriptomes	Metatranscriptomic samples from the Amazon river-sea continuum sampling effort. Samples from the Amazon river.	ANACONDAS	237345	39	NCBI
Amazon PolyA Metatranscriptomes	PolyA-enriched metatranscriptomes samples from the Amazon river-sea continuum sampling effort.	ANACONDAS	237346	22	NCBI, BCO-DMO, R2R, SAMOS, WHOI

#### Datasets included in the database

Regardless of the scientific question, critical changes in an ecosystem can only be understood within the appropriate historical context that allows the investigation of long-term trends. Thus, the first phase of Planet Microbe focused on historical time-series oceanographic ‘omics datasets including the Hawaii Ocean Time-series (HOT) (19) and Bermuda Atlantic Time-series (BATS) (22). These foundational data sets have a long history of data-rich sampling efforts.

While these time-series datasets allow for a comprehensive view of a particular ecosystem of interest, our understanding of oceanic microbial population has recently been expanded by large-scale worldwide sampling expeditions. Therefore, Planet Microbe also includes the ‘omics datasets from Global Ocean Sampling Expedition (GOS) (23), TARA Oceans (27), and Arctic Expeditions (28). We also included the Ocean Sampling Day (OSD) (29), a project of the Genomic Observatories Network that involved a simultaneous one-day sampling campaign of the world's oceans in the summer solstice of 2014.

Finally, Planet Microbe includes datasets exploring two ecosystems of particular interest. First, this project included the Amazon continuum dataset (ANACONDAS) (24,25), aiming to provide an understanding of the microbial population across a fresh-water to a sea-water gradient. Finally, we included deep seawater samples from the Center for Dark Energy Biosphere Investigations (C-DEBI) (26)).

### Standardization of attributes

#### Unified terminology bridging various data annotation frameworks

In NCBI BioSample and other data resources, sample attributes are written into single-slot text fields that do not strictly enforce any naming or style conventions. In BCO-DMO, a consistent vocabulary is used across datasets, however, these terms are specific to their data model. Similarly, OSD relies on the EU FP7 Project MicroB3 for the development of its vocabulary, metadata collection protocols, and processing workflows. While these efforts make data interoperable within a given collection effort, these vocabularies are not consistent across data sources and projects.

For these reasons, Planet Microbe uses a unified semantic layer to make the datasets, data sources, and units interoperable. Attributes described in the Minimum Information about any (x) Sequence (MIxS) water checklist (6) were annotated using terms from an application ontology that imports terms from various OBO (Open Biological and Biomedical Ontologies Foundry) ontologies as the Environment Ontology (ENVO) (38–40).

The application ontology called Planet Microbe Ontology (PMO) was constructed using the ontology-development-kit https://github.com/INCATools/ontology-development-kit and is available from https://github.com/hurwitzlab/planet-microbe-ontology.

To be integrated into Planet Microbe, a minimum set of information about a sample was required: BioSample ID, sample latitude, sample longitude, sample depth, and sample collection date, or date-time.

#### Annotation with MIxS mandated ENVO terms

As many of the Planet Microbe datasets made use of the MIxS Water version 4 checklist during submission to NCBI, they were mostly annotated with ENVO biome, environmental feature, and environmental material terms. However, leveraging these annotations proved difficult due to misannotations (i.e. the use of terms from an incorrect ENVO hierarchy), the use of deprecated terms, or the use of term labels that do not correspond to actual ENVO terms. Manual curation of these three terms was therefore performed on our samples in order to provide consistent annotation and enable data to be searchable based on these environmental descriptors.

#### Unit harmonization

Latitude and longitude coordinates were standardized to the format of Decimal Degrees. Additionally, when applicable, attributes described in the MIxS water checklist were annotated using the Unit Ontology (UO) from the OBO foundry (41). Upon upload into the Planet Microbe database, these annotations were used to automatically convert attribute measurements from the original source units to the units recommended by the MIxS Water checklist. Finally, for each sample, dates and date-time formats were described using the Frictionless Data package date format, allowing interoperability through the different formats.

#### Source tracking

Planet Microbe aims to collect environmental contextual metadata about samples from different sources. Therefore, each sample attribute was annotated with a source URL or DOI allowing users to track the source of the information back to its original resource.

### Web-platform implementation

The Planet Microbe web architecture consists of the frontend user interface and back-end API (Figure 2). The frontend is written in Elm (42) and the backend API is written in Node.js (43). The database is implemented in PostgreSQL via Python load scripts and Frictionless Data libraries. Authentication (OAUTH2), cloud storage (CyVerse Data Store), and computation (TACC Stampede2) are enabled by the TACC Cloud API System (TAPIS).

The code is available on GitHub from https://github.com/hurwitzlab/planet-microbe-app. Documentation is available in Gitbook accessible through the web interface (see the documentation tab) or directly from https://hurwitzlab.gitbook.io/planet-microbe-documentation/.

The Planet Microbe home page (https://www.planetmicrobe.org) gives an overview of the project's latest developments, vision, and aims. The navigation bar allows users to access the main components of the Planet Microbe platform. The Planet Microbe user interface is divided into three main sections: ‘Browse’, ‘Search’ and ‘Analyze’. The ‘Browse’ section gives an overview of the various marine metagenomic projects integrated into Planet microbe and provides access to our FTP access point. The ‘Search’ section constitutes the main search interface for the samples integrated into Planet Microbe. The ‘Analyze’ section allows users to run applications on datasets included in Planet Microbe or their own datasets.

### Leveraging community cyberinfrastructure for data analysis

Planet Microbe is a ‘powered by CyVerse’ project and leverages CyVerse services such as OAuth2 authentication and the CyVerse Data Store for the storage of datasets and analysis results (i.e. cloud-based storage optimized for large datasets that are freely accessible through multiple interfaces) (31). Additionally, Planet Microbe leverages HPC resources from the Texas Advanced Computing Center (TACC Stampede2) for computation. Finally, access to CyVerse services is enabled by the TACC Cloud API System (TAPIS) (44).

Planet Microbe allows users to run applications (‘Apps’) on the Planet Microbe datasets or a user's own datasets in the CyVerse Data Store. In addition to TAPIS/Stampede2, Planet Microbe uses a custom Node.js service to deploy jobs on a local server (https://github.com/hurwitzlab/plan-b). The provenance of primary data, derived files, and analyses are tracked in CyVerse by keeping all files in the analysis directory, along with data products and a log file. Job inputs and parameters are saved by TAPIS.

## DISCUSSION

Understanding complex ocean systems requires the integration of biological (particularly microbial) processes with characteristics associated with the environment, to understand environmental resilience and adaptive potential to change. These complex systems can only be understood given extensive sampling efforts that include data from diverse domains of ecosystem science (i.e. ‘omics, microbiology, biogeochemistry, as well as physical, chemical, and biological oceanography). To enable integrated research across these disciplines, systems compiling and interlinking data while allowing their analysis using high-performance computer architectures are needed. Planet Microbe is a web-based platform aiming to centralize and standardize contextual data associated with major marine ‘omic datasets.

### Centralizing and aggregating datasets

Large-scale ‘omics repositories like NCBI (5), MG-RAST (10) or JGI (45) allow for access to ‘omics datasets, while oceanographic data repositories like BCO-DMO allow researchers to share important sample contextual data. However, we believe Planet Microbe and other aggregation and curation efforts to be critical to enhance the reuse of these distributed datasets. Planet Microbe aims to centralize and link data sets from a variety of resources, making them interoperable with one another. To be included in Planet Microbe, the dataset needs to be open-source and available in a public sequence repository that is affiliated with the International Nucleotide Sequence Database Collaboration (INSDC, www.insdc.org). Because the project is collecting data from a large number of sources, an effort has been made to provide a source URL or DOI for each datum accessible in Planet Microbe.

Importantly, our approach does not aim to impose a new proprietary ingestion standard but instead preserves the original dataset as published (along with supplemental curation and correction efforts). With this in mind, we made datasets interoperable by applying a unified semantic layer to standardize, rather than rename existing dataset attributes. Similarly, Planet Microbe can upload data with attributes collected in a variety of units, as unit conversions are computed automatically during upload into our database.

### Need for large-scale intercalibration efforts

The work presented here brings together a large number of marine ‘omics samples. However, it is important to note that these datasets were prepared using different methods (i.e. sample preservation, extraction, amplification, and sequencing) and therefore have unique biases that make them quantitatively incomparable without correction (46). Future efforts are needed to develop and describe robust community-accepted methods for sample collection, sample size fractionation, filtration, and quantitation using mock community spike-ins for creating cross-comparable ‘omics datasets. Further, bioinformatics protocols and pipelines require standardization to make taxonomic and functional annotations comparable across projects, and statistical methods should be made easily accessible to account for biases between datasets. Concerted community-driven efforts are required to allow datasets to be more effectively used together to better elucidate global questions on microbial driven biogeochemical processes in the ocean.

### Ongoing development

Planet Microbe is currently in its first release and is expected to be updated regularly, as new large-scale marine ‘omics projects are made available. Moreover, future development will include consistent taxonomic and functional annotation of the datasets currently available in Planet Microbe and the extension of our system to deploy new search capabilities leveraging these annotations.

Future work will also include the integration of additional data types currently not supported in our system, and in particular, the integration of satellite-derived measurements. Finally, we plan to develop resources and tools to help scientists to harmonize their datasets with the larger collection of data in Planet Microbe, or prepare and plan future data collection expeditions using standardized terminology from OBO ontologies.

## DATA AVAILABILITY

Planet Microbe is freely accessible at https://www.planetmicrobe.org. Documentation is available in Gitbook accessible through the web interface (see the documentation tab) or directly from https://hurwitzlab.gitbook.io/planet-microbe-documentation/. The code is available on GitHub from https://github.com/hurwitzlab/planet-microbe-app.

## References

[1] Venter J.C., Remington K., Heidelberg J.F., Halpern A.L., Rusch D., Eisen J.A., Wu D., Paulsen I., Nelson K.E., Nelson W. et al. Environmental genome shotgun sequencing of the Sargasso Sea. Science. 2004; 304:66–74. 15001713 10.1126/science.1093857

[2] Sunagawa S., Coelho L.P., Chaffron S., Kultima J.R., Labadie K., Salazar G., Djahanschiri B., Zeller G., Mende D.R., Alberti A. et al. Structure and function of the global ocean microbiome. Science. 2015; 348:1261359. 25999513 10.1126/science.1261359

[3] Leinonen R., Sugawara H., Shumway M. The sequence read archive. Nucleic Acids Res. 2011; 39:D19–D21. 21062823 10.1093/nar/gkq1019 PMC3013647

[4] Chandler C.L., Groman R.C., Allison M.D., Wiebe P.H., Glover D.M., Gegg S.R. Effective management of ocean biogeochemistry and ecological data: the BCO-DMO story. EGU General Assembly Conference Abstracts. 2012; 14:1258.

[5] Barrett T., Clark K., Gevorgyan R., Gorelenkov V., Gribov E., Karsch-Mizrachi I., Kimelman M., Pruitt K.D., Resenchuk S., Tatusova T. et al. BioProject and BioSample databases at NCBI: facilitating capture and organization of metadata. Nucleic Acids Res. 2012; 40:D57–D63. 22139929 10.1093/nar/gkr1163 PMC3245069

[6] Yilmaz P., Kottmann R., Field D., Knight R., Cole J.R., Amaral-Zettler L., Gilbert J.A., Karsch-Mizrachi I., Johnston A., Cochrane G. et al. Minimum information about a marker gene sequence (MIMARKS) and minimum information about any (x) sequence (MIxS) specifications. Nat. Biotechnol. 2011; 29:415–420. 21552244 10.1038/nbt.1823 PMC3367316

[7] Mitchell A.L., Scheremetjew M., Denise H., Potter S., Tarkowska A., Qureshi M., Salazar G.A., Pesseat S., Boland M.A., Hunter F.M.I. et al. EBI metagenomics in 2017: enriching the analysis of microbial communities, from sequence reads to assemblies. Nucleic Acids Res. 2018; 46:D726–D735. 29069476 10.1093/nar/gkx967 PMC5753268

[8] Bernstein M.N., Doan A., Dewey C.N. MetaSRA: normalized human sample-specific metadata for the sequence read archive. Bioinforma. Oxf. Engl. 2017; 33:2914–2923. 10.1093/bioinformatics/btx334 PMC5870770 28535296

[9] Corrêa F.B., Saraiva J.P., Stadler P.F., da Rocha U.N. TerrestrialMetagenomeDB: a public repository of curated and standardized metadata for terrestrial metagenomes. Nucleic Acids Res. 2020; 48:D626–D632. 31728526 10.1093/nar/gkz994 PMC7145636

[10] Meyer F., Bagchi S., Chaterji S., Gerlach W., Grama A., Harrison T., Paczian T., Trimble W.L., Wilke A. MG-RAST version 4—lessons learned from a decade of low-budget ultra-high-throughput metagenome analysis. Brief. Bioinform. 2019; 20:1151–1159. 29028869 10.1093/bib/bbx105 PMC6781595

[11] Rodriguez-Tomé P., Stoehr P.J., Cameron G.N., Flores T.P. The European Bioinformatics Institute (EBI) databases. Nucleic Acids Res. 1996; 24:6–12. 8594602 10.1093/nar/24.1.6 PMC145572

[12] Klemetsen T., Raknes I.A., Fu J., Agafonov A., Balasundaram S.V., Tartari G., Robertsen E., Willassen N.P. The MAR databases: development and implementation of databases specific for marine metagenomics. Nucleic Acids Res. 2018; 46:D692–D699. 29106641 10.1093/nar/gkx1036 PMC5753341

[13] Pasolli E., Schiffer L., Manghi P., Renson A., Obenchain V., Truong D.T., Beghini F., Malik F., Ramos M., Dowd J.B. et al. Accessible, curated metagenomic data through ExperimentHub. Nat. Methods. 2017; 14:1023–1024. 29088129 10.1038/nmeth.4468 PMC5862039

[14] Sweetlove M., Gan Y.M., Murray A., de Putte A.V. The microbial Antarctic resource system: integrating discoverability and preservation of environmentally-annotated microbial ’omics data. Biodivers. Inf. Sci. Stand. 2019; 3:e37499.

[15] Villar E., Vannier T., Vernette C., Lescot M., Cuenca M., Alexandre A., Bachelerie P., Rosnet T., Pelletier E., Sunagawa S. et al. The Ocean Gene Atlas: exploring the biogeography of plankton genes online. Nucleic Acids Res. 2018; 46:W289–W295. 29788376 10.1093/nar/gky376 PMC6030836

[16] Altschul S.F., Gish W., Miller W., Myers E.W., Lipman D.J. Basic local alignment search tool. J. Mol. Biol. 1990; 215:403–410. 2231712 10.1016/S0022-2836(05)80360-2

[17] Bryant J.A., Aylward F.O., Eppley J.M., Karl D.M., Church M.J., DeLong E.F. Wind and sunlight shape microbial diversity in surface waters of the North Pacific Subtropical Gyre. ISME J. 2016; 10:1308–1322. 26645474 10.1038/ismej.2015.221 PMC5029195

[18] Karl D.M., Church M.J. Microbial oceanography and the Hawaii Ocean Time-series programme. Nat. Rev. Microbiol. 2014; 12:699–713. 25157695 10.1038/nrmicro3333

[19] Karl D.M., Lukas R. The Hawaii Ocean Time-series (HOT) program: background, rationale and field implementation. Deep Sea Res. Part II Top. Stud. Oceanogr. 1996; 43:129–156.

[20] Luo E., Eppley J.M., Romano A.E., Mende D.R., DeLong E.F. Double-stranded DNA virioplankton dynamics and reproductive strategies in the oligotrophic open ocean water column. ISME J. 2020; 14:1304–1315. 32060418 10.1038/s41396-020-0604-8 PMC7174320

[21] Mende D.R., Bryant J.A., Aylward F.O., Eppley J.M., Nielsen T., Karl D.M., DeLong E.F. Environmental drivers of a microbial genomic transition zone in the ocean's interior. Nat. Microbiol. 2017; 2:1367–1373. 28808230 10.1038/s41564-017-0008-3

[22] Michaels A.F., Knap A.H. Overview of the U.S. JGOFS Bermuda Atlantic Time-series Study and the Hydrostation S program. Deep Sea Res. Part II Top. Stud. Oceanogr. 1996; 43:157–198.

[23] Rusch D.B., Halpern A.L., Sutton G., Heidelberg K.B., Williamson S., Yooseph S., Wu D., Eisen J.A., Hoffman J.M., Remington K. et al. The sorcerer II global ocean sampling Expedition: Northwest atlantic through Eastern tropical Pacific. PLoS Biol. 2007; 5:e77. 17355176 10.1371/journal.pbio.0050077 PMC1821060

[24] Satinsky B.M., Zielinski B.L., Doherty M., Smith C.B., Sharma S., Paul J.H., Crump B.C., Moran M.A. The Amazon continuum dataset: quantitative metagenomic and metatranscriptomic inventories of the Amazon River plume, June 2010. Microbiome. 2014; 2:17. 24883185 10.1186/2049-2618-2-17 PMC4039049

[25] Satinsky B.M., Fortunato C.S., Doherty M., Smith C.B., Sharma S., Ward N.D., Krusche A.V., Yager P.L., Richey J.E., Moran M.A. et al. Metagenomic and metatranscriptomic inventories of the lower Amazon River, May 2011. Microbiome. 2015; 3:39. 26353777 10.1186/s40168-015-0099-0 PMC4564970

[26] Orcutt B.N., Sylvan J.B., Knab N.J., Edwards K.J. Microbial ecology of the dark ocean above, at, and below the Seafloor. Microbiol. Mol. Biol. Rev. 2011; 75:361–422. 21646433 10.1128/MMBR.00039-10 PMC3122624

[27] Pesant S., Not F., Picheral M., Kandels-Lewis S., Bescot N.L., Gorsky G., Iudicone D., Karsenti E., Speich S., Troublé R. et al. Open science resources for the discovery and analysis of Tara Oceans data. Sci. Data. 2015; 2:150023. 26029378 10.1038/sdata.2015.23 PMC4443879

[28] Gregory A.C., Zayed A.A., Conceição-Neto N., Temperton B., Bolduc B., Alberti A., Ardyna M., Arkhipova K., Carmichael M., Cruaud C. et al. Marine DNA viral macro- and microdiversity from pole to pole. Cell. 2019; 177:1109–1123. 31031001 10.1016/j.cell.2019.03.040 PMC6525058

[29] Kopf A., Bicak M., Kottmann R., Schnetzer J., Kostadinov I., Lehmann K., Fernandez-Guerra A., Jeanthon C., Rahav E., Ullrich M. et al. The ocean sampling day consortium. GigaScience. 2015; 4:27. 26097697 10.1186/s13742-015-0066-5 PMC4473829

[30] Goff S.A., Vaughn M., McKay S., Lyons E., Stapleton A.E., Gessler D., Matasci N., Wang L., Hanlon M., Lenards A. et al. The iPlant Collaborative: Cyberinfrastructure for plant biology. Front. Plant Sci. 2011; 2:34. 22645531 10.3389/fpls.2011.00034 PMC3355756

[31] Merchant N., Lyons E., Goff S., Vaughn M., Ware D., Micklos D., Antin P. The iPlant Collaborative: Cyberinfrastructure for enabling data to discovery for the life sciences. PLOS Biol. 2016; 14:e1002342. 26752627 10.1371/journal.pbio.1002342 PMC4709069

[32] Kim D., Song L., Breitwieser F.P., Salzberg S.L. Centrifuge: rapid and sensitive classification of metagenomic sequences. Genome Res. 2016; 26:1721–1729. 27852649 10.1101/gr.210641.116 PMC5131823

[33] Choi I., Ponsero A.J., Bomhoff M., Youens-Clark K., Hartman J.H., Hurwitz B.L. Libra: scalable k-mer–based tool for massive all-vs-all metagenome comparisons. GigaScience. 2019; 8:giy165. 10.1093/gigascience/giy165 PMC6354030 30597002

[34] Ondov B.D., Treangen T.J., Melsted P., Mallonee A.B., Bergman N.H., Koren S., Phillippy A.M. Mash: fast genome and metagenome distance estimation using MinHash. Genome Biol. 2016; 17:132. 27323842 10.1186/s13059-016-0997-x PMC4915045

[35] Boeuf D., Edwards B.R., Eppley J.M., Hu S.K., Poff K.E., Romano A.E., Caron D.A., Karl D.M., DeLong E.F. Biological composition and microbial dynamics of sinking particulate organic matter at abyssal depths in the oligotrophic open ocean. Proc. Natl. Acad. Sci. U.S.A. 2019; 116:11824–11832. 31127042 10.1073/pnas.1903080116 PMC6575173

[36] Fontanez K.M., Eppley J.M., Samo T.J., Karl D.M., DeLong E.F. Microbial community structure and function on sinking particles in the North Pacific subtropical gyre. Front. Microbiol. 2015; 6:469. 26042105 10.3389/fmicb.2015.00469 PMC4436931

[37] Rocca-Serra P., Sansone S.-A. Experiment design driven FAIRification of omics data matrices, an exemplar. Sci. Data. 2019; 6:271. 31831744 10.1038/s41597-019-0286-0 PMC6908569

[38] Buttigieg P.L., Morrison N., Smith B., Mungall C.J., Lewis S.E. the ENVO Consortium The environment ontology: contextualising biological and biomedical entities. J. Biomed. Semant. 2013; 4:43. 10.1186/2041-1480-4-43 PMC3904460 24330602

[39] Buttigieg P.L., Pafilis E., Lewis S.E., Schildhauer M.P., Walls R.L., Mungall C.J. The environment ontology in 2016: bridging domains with increased scope, semantic density, and interoperation. J. Biomed. Semant. 2016; 7:57. 10.1186/s13326-016-0097-6 PMC5035502 27664130

[40] Smith B., Ashburner M., Rosse C., Bard J., Bug W., Ceusters W., Goldberg L.J., Eilbeck K., Ireland A., Mungall C.J. et al. The OBO Foundry: coordinated evolution of ontologies to support biomedical data integration. Nat. Biotechnol. 2007; 25:1251–1255. 17989687 10.1038/nbt1346 PMC2814061

[41] Gkoutos G.V., Schofield P.N., Hoehndorf R. The Units Ontology: a tool for integrating units of measurement in science. Database. 2012; 2012:bas033. 23060432 10.1093/database/bas033 PMC3468815

[42] Czaplicki E. Elm: concurrent frp for functional guis. 2012; Sr. Thesis, Harv. Univ.

[43] Teixeira P. Professional Node.js: building Javascript based scalable software. 2012; John Wiley & Sons.

[44] Dooley R., Brandt S.R., Fonner J. The Agave Platform: An Open, Science-as-a-Service Platform for Digital Science. Proceedings of the Practice and Experience on Advanced Research Computing. 2018; Pittsburgh Association for Computing Machinery 1–8.PEARC ’18.

[45] Kyrpides N.C. Genomes OnLine Database (GOLD 1.0): a monitor of complete and ongoing genome projects world-wide. Bioinformatics. 1999; 15:773–774. 10498782 10.1093/bioinformatics/15.9.773

[46] McLaren M.R., Willis A.D., Callahan B.J. Consistent and correctable bias in metagenomic sequencing experiments. eLife. 2019; 8:e46923. 31502536 10.7554/eLife.46923 PMC6739870

